# Supplementary material for: The role of risk communication in public health interventions. An analysis of risk communication for a community quarantine in Germany to curb the SARS-CoV-2 pandemic
Source: PLoS One. 2021 Aug 13;16(8):e0256113. doi: 10.1371/journal.pone.0256113 (PMC8362954; doi:10.1371/journal.pone.0256113)
Supplement: S1 Table — The data of the questionnaire are listed in detail (whole numbers and percent) for every question, except for the open-ended questions. (PDF) [file pone.0256113.s001.pdf]

S1 Table. Results in detail.

The data of the questionnaire are listed in detail (whole numbers and percent) for every question, except for the open-ended questions.

1.	age distribution	18 - 29	30 - 39	40 - 49	50 - 59	60 - 69	over 70
		14 (4.8%)	35 (12.1%)	43 (14.9%)	61 (21.1%)	71 (24.6%)	44 (22.1%)
2.	gender	male	female				
		132 (45.7%)	157 (54.3%)				
3.	number of persons per household	N/A	1	more than 1			
		1 (0.4%)	40 (13.8%)	248 (85.8%)			
4.	use of media before quarantine	N/A	never	seldom	occasional	frequent	very frequent
	newspaper	54 (18.7%)	73 (25.3%)	32 (11.1%)	52 (18.0%)	24 (8.3%)	54 (18.7%)
	television	9 (3.1%)	6 (2.1%)	24 (8.3%)	62 (21.5%)	63 (21.8%)	125 (43.3%)
	radio	26 (9.0%)	27 (9.3%)	39 (13.5%)	72 (24.9%)	44 (15.2%)	81 (28.0%)
	internet	42 (14.5%)	63 (21.8%)	31 (10.7%)	36 (12.5%)	53 (18.3%)	64 (22.2%)
	authorities	69 (23.9%)	108 (37.4%)	35 (12.1%)	36 (12.5%)	20 (6.9%)	21 (7.3%)
	social media	68 (23.5%)	106 (36.7%)	36 (12.5%)	35 (12.1%)	20 (6.9%)	24 (8.3%)
	partner/spouse	79 (27.3%)	38 (13.2%)	29 (10.0%)	37 (12.8%)	45 (15.6%)	61 (21.1%)

	physician	68 (23.5%)	142 (49.1%)	37 (12.8%)	29 (10.0%)	6 (2.1%)	7 (2.4%)
	pharmacy	74 (25.6%)	164 (56.8%)	30 (10.4%)	11 (3.8%)	6 (2.1%)	4 (1.4%)
	neighbour	57 (19.7%)	104 (36.0%)	57 (19.7%)	40 (13.8%)	13 (4.5%)	18 (6.2%)
5.	use of media during quarantine						
	newspaper	57 (19.7%)	105 (36.3%)	28 (9.7%)	37 (12.8%)	23 (8.0%)	39 (13.5%)
	television	13 (4.5%)	6 (2.1%)	19 (6.6%)	52 (18.0%)	62 (21.5%)	137 (47.4%)
	radio	38 (13.2%)	29 (10.0%)	30 (10.4%)	67 (23.2%)	40 (13.8%)	85 (29.4%)
	internet	46 (16.0%)	57 (19.7%)	22 (7.6%)	41 (14.2%)	39 (13.5%)	84 (29.1%)
	authorities	71 (24.6%)	98 (34.0%)	36 (12.5%)	28 (9.7%)	25 (8.7%)	31 (10.7%)
	social media	77 (26.7%)	100 (34.6%)	32 (11.1%)	22 (7.6%)	26 (9.0%)	32 (11.1%)
	partner/spouse	84 (29.1%)	40 (13.8%)	21 (7.3%)	41 (14.2%)	31 (10.7%)	72 (25.0%)
	physician	78 (27.0%)	133 (46.0%)	41 (14.2%)	26 (9.0%)	6 (2.1%)	5 (1.7%)
	pharmacy	88 (30.5%)	161 (55.7%)	25 (8.7%)	11 (3.8%)	3 (1.0%)	1 (0.4%)
	neighbour	68 (23.5%)	106 (36.7%)	50 (17.3%)	37 (12.8%)	15 (5.2%)	13 (4.5%)
6.	information by authorities						
	internet	83 (28.7%)	76 (26.3%)	31 (10.7%)	39 (13.5%)	25 (8.7%)	35 (12.1%)
	leaflet	30 (10.4%)	22 (7.6%)	58 (20.1%)	106 (36.7%)	35 (12.1%)	38 (13.2%)
	radio	75 (26.0%)	62 (21.5%)	50 (17.3%)	38 (13.2%)	29 (10.0%)	35 (12.1%)

home visits	95 (32.9%)	170 (58.8%)	16 (5.5%)	6 (2.1%)	0 (0.0%)	2 (0.7%)
loudspeaker announcements	19 (6.6%)	20 (6.9%)	65 (22.5%)	91 (31.5%)	39 (13.5%)	55 (19.0%)
7. level of information	N/A	very poor	poor	moderate	good	very good
	8 (2.8%)	26 (9.0%)	44 (15.2%)	90 (31.1%)	57 (19.7%)	64 (22.2%)
8. level of concern	N/A	very low	low	moderate	high	very high
	8 (2.8%)	33 (11.4%)	48 (16.6%)	72 (24.9%)	52 (18.0%)	76 (26.3%)
9. level of concern (specific topics)						
personal physical health	7 (2.4%)	37 (12.8%)	60 (20.8%)	52 (18.0%)	39 (13.5%)	94 (32.5%)
family's health	10 (3.5%)	8 (2.8%)	22 (7.6%)	37 (12.8%)	47 (16.3%)	165 (57.1%)
personal mental health	22 (7.6%)	73 (25.3%)	40 (13.8%)	60 (20.8%)	30 (10.4%)	64 (22.2%)
nation's economic stability	25 (8.7%)	47 (16.3%)	34 (11.8%)	69 (23.9%)	49 (17.0%)	65 (22.5%)
personal financial stability	19 (6.6%)	70 (24.2%)	47 (16.3%)	50 (17.3%)	39 (13.5%)	64 (22.2%)
nation's political stability	28 (9.7%)	74 (25.6%)	35 (12.1%)	58 (20.1%)	53 (18.3%)	41 (14.2%)
personal employment security	51 (17.7%)	109 (37.7%)	24 (8.3%)	27 (9.3%)	22 (7.6%)	56 (19.4%)
11. communication with authorities	N/A	never	seldom	occasional	frequent	very frequent
telephone hotline	75 (26.0%)	106 (36.7%)	40 (13.8%)	37 (12.8%)	19 (6.6%)	12 (4.2%)
telephone	95	139	25	14	7	9

	appointment	(32.9%)	(48.1%)	(8.7%)	(4.8%)	(2.4%)	(3.1%)
	online chat	113	159	9	4	2	2
		(39.1%)	(55.0%)	(3.1%)	(1.4%)	(0.7%)	(0.7%)
12.	acceptance of quarantine	N/A	yes	no			
		9	212	68			
		(3.1%)	(73.4%)	(23.5%)			
13.	compliance with quarantine	N/A	everyone	the majority	half of N.a.R.	few	nobody
		13	20	217	15	2	3
		(4.5%)	(6.9%)	(75.1%)	(5.2%)	(7.3%)	(1.0%)
14.	approval of non-compliance	N/A	yes	no			
		7	27	255			
		(2.4%)	(9.3%)	(88.2%)			
15.	avoidance of places/persons after quarantine	N/A	yes	no			
		11	116	162			
		(3.8%)	(40.1%)	(56.1%)			
