# Supplementary material for: The role of risk communication in public health interventions. An analysis of risk communication for a community quarantine in Germany to curb the SARS-CoV-2 pandemic
Source: PLoS One. 2021 Aug 13;16(8):e0256113. doi: 10.1371/journal.pone.0256113 (PMC8362954; doi:10.1371/journal.pone.0256113)
Supplement: S1 File — The questionnaire which was used to obtain the data. (PDF) [file pone.0256113.s002.pdf]

S1 File. Questionnaire. The questionnaire which was used to obtain the data.

FRAGEBOGEN

RISIKOKOMMUNIKATION |

Die Rolle von Risikokommunikation im Pandemiemanagement in der Analyse des Ausbruchsgeschehen in Neustadt am Rennsteig

FORSCHUNGSGRUPPE PANDEMIEMANAGEMENT

11. Mai 2020

PD Dr. med. Dr. phil. **Petra Dickmann** MA

Klinik für Anästhesie und Intensivmedizin (KAI)

Leitung und Koordination der

Forschungsgruppe ***Pandemiemanagement***

Uniklinik Jena (UKJ)

pdickmann@dickmann-drc.com

petra.dickmann@med.uni-jena.de

Fragebogen Risikokommunikation im Pandemiemanagement - Case Study Das Ausbruchsgeschehen und die Quarantäne in Neustadt am Renn-steig

PD Dr. Dr. Petra Dickmann, Leitung

Dr. Wibke Wetzker, Post-Doc

cand. med. Juliane Scholz, Stud. Hilfskraft und Doktorandin

cand. med. Annika Licht, Stud. Hilfskraft

Bitte füllen Sie den Fragebogen nur aus, wenn Sie über 18 Jahre alt sind.

Fragebogen

I. Allgemeine Angaben

1. Alter: _ _ _ Jahre

2. Geschlecht

- ◻ männlich
- ◻ weiblich
- ◻ divers

3. Wie viele Personen leben in Ihrem Haushalt?

- ◻ alleinstehend
- ◻ mehr als 1 Person

II. Informationen

4. Welche Medien oder Personen haben Sie **vor** der Anordnung der häuslichen Quarantäne (bis 22. März 2020) in Neustadt genutzt, um sich über die Coronavirus-Pandemie zu informieren? (Bitte Wert zwischen 1 und 5 ankreuzen)

- ◻ Tageszeitungen

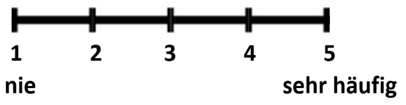

- □ Fernsehen

  1 2 3 4 5
  nie sehr häufig

- □ Radio

  1 2 3 4 5
  nie sehr häufig

- □ Internet (allgemein)

  1 2 3 4 5
  nie sehr häufig

- □ Offizielle Behörden (online, z.B. RKI, BZgA, WHO etc.)

  1 2 3 4 5
  nie sehr häufig

- □ Social Media

  1 2 3 4 5
  nie sehr häufig

- □ Mein/e Lebenspartner/in

  1 2 3 4 5
  nie sehr häufig

- □ Arzt

  1 2 3 4 5
  nie sehr häufig

- □ Apotheke oder anderes medizinisches Personal

  1 2 3 4 5
  nie sehr häufig

- □ Nachbarn

  1 2 3 4 5
  nie sehr häufig

- □ Andere

5. Welche Medien oder Personen haben Sie **während** der häuslichen Quarantäne (ab 22. März 2020) in Neustadt genutzt, um sich über die Coronavirus-Pandemie zu informieren? (Bitte Wert zwischen 1 und 5 ankreuzen)

- ☐ Tageszeitungen

  1 2 3 4 5
  nie sehr häufig

- ☐ Fernsehen

  1 2 3 4 5
  nie sehr häufig

- ☐ Radio

  1 2 3 4 5
  nie sehr häufig

- ☐ Internet (allgemein)

  1 2 3 4 5
  nie sehr häufig

- ☐ Offizielle Behörden (online, z.B. RKI, BZgA, WHO etc.)

  1 2 3 4 5
  nie sehr häufig

- ☐ Social Media

  1 2 3 4 5
  nie sehr häufig

- ☐ Mein/e Lebenspartner/in

  1 2 3 4 5
  nie sehr häufig

- ☐ Arzt

  1 2 3 4 5
  nie sehr häufig

- ☐ Apotheke oder anderes medizinisches Personal

  1 2 3 4 5
  nie sehr häufig

- ☐ Nachbarn

  1 2 3 4 5
  nie sehr häufig

□ Andere

6. Wie haben Sie Informationen **während der Quarantäne** von den örtlichen Behörden erhalten? (Bitte Wert zwischen 1 und 5 ankreuzen)

□ Internet

1 2 3 4 5
nie sehr häufig

□ Flyer

1 2 3 4 5
nie sehr häufig

□ Radio

1 2 3 4 5
nie sehr häufig

□ Hausbesuche

1 2 3 4 5
nie sehr häufig

□ Durchsagen

1 2 3 4 5
nie sehr häufig

□ Andere

7. Wie gut haben Sie sich **während der Quarantäne** informiert gefühlt? (Bitte Wert zwischen 1 und 5 ankreuzen)

1 2 3 4 5
gar nicht gut sehr gut

□ Es gab viel zu viele Informationen

8. Welche Informationen hätten Sie sich **während der Quarantäne** gewünscht?

9. Wie besorgt waren Sie **während der Quarantäne**? (Bitte Wert zwischen 1 und 5 ankreuzen)

1 2 3 4 5
gar nicht sehr

10. Worüber haben Sie sich Sorgen gemacht? (Bitte Wert zwischen 1 und 5 ankreuzen)

Meine eigene Gesundheit

1 2 3 4 5
gar nicht sehr

Die Gesundheit meiner Familie

1 2 3 4 5
gar nicht sehr

Mein psychisches Wohlergehen

1 2 3 4 5
gar nicht sehr

Die wirtschaftliche Stabilität meines Landes (Rezession)

1 2 3 4 5
gar nicht sehr

Meine persönliche finanzielle Situation

1 2 3 4 5
gar nicht sehr

Die politische Stabilität meines Landes

1 2 3 4 5
gar nicht sehr

Meine Arbeitsplatzsicherheit

1 2 3 4 5
gar nicht sehr

Andere

II. Kommunikation mit den Behörden

11. Welche Möglichkeiten des Kontakts zu Behörden haben Sie genutzt? (Bitte Wert zwischen 1 und 5 ankreuzen)

- ▫ Telefonhotline

  1 2 3 4 5
  nie sehr häufig

- ▫ Telefontermin

  1 2 3 4 5
  nie sehr häufig

- ▫ Online Chat

  1 2 3 4 5
  nie sehr häufig

- ▫ Andere
- ▫ Keine

III. Koordination

12. Haben Sie die Quarantäne als der Lage angemessen empfunden?

- ▫ Ja
- ▫ Nein

13. Wie groß war Ihrer Meinung nach der Anteil der Bewohner von Neustadt, der sich an die Quarantäne gehalten hat? (Bitte Wert zwischen 1 und 5 ankreuzen)

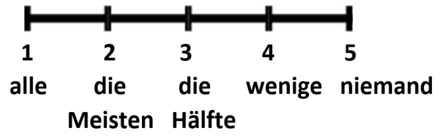

14. Können Sie verstehen, dass sich einige Personen nicht an die Quarantäne gehalten haben?

- ▫ Ja
- ▫ Nein

Wenn ja, warum?

15. Haben Sie nach dem Ende der Quarantäne bestimmte Orte oder Personengruppen gemieden, weil Sie von einem hohen Ansteckungspotential ausgegangen sind?

- ▫ Ja
- ▫ Nein

Wenn Ja, welche:

16. Wenn in anderen Regionen eine häusliche Quarantäne für eine ganze Gemeinde oder Stadt angeordnet wird, was empfehlen Sie den lokalen Behörden an Maßnahmen:

1. ______________________________

2. ______________________________

3. ____

____
