# Supplementary material for: The role of risk communication in public health interventions. An analysis of risk communication for a community quarantine in Germany to curb the SARS-CoV-2 pandemic
Source: PLoS One. 2021 Aug 13;16(8):e0256113. doi: 10.1371/journal.pone.0256113 (PMC8362954; doi:10.1371/journal.pone.0256113)
Supplement: S2 File — The information leaflet was given out to the population of Neustadt am Rennsteig during quarantine. It explains how postal deliveries, waste disposal and the working situation were handled. (PDF) [file pone.0256113.s003.pdf]

S2 File. Information leaflet.

The information leaflet was given out to the population of Neustadt am Rennsteig during quarantine. It explains how postal deliveries, waste disposal and the working situation were handled.

Infoblatt für Neustadt

Liebe Bürgerinnen und Bürger von Neustadt,

die schnelle Ausbreitung des „Coronavirus“ in Ihrer Ortschaft Neustadt a. Rstg. stellt uns vor neue Herausforderungen. Ich möchte Sie alle bitten, die für Neustadt erlassenen Regeln einzuhalten.
Die Vermeidung direkter sozialer Kontakte ist in dieser Situation aktuell das Wichtigste. Denken Sie in erster Linie an Ihre eigene Gesundheit, an die Ihrer Familie sowie an Ihre Mitmenschen und vor allem an jene, welche bereits im höheren Alter sind oder Vorerkrankungen haben.

Die gegenwärtige Situation wirft sicherlich viele Fragen auf.
Gern können Sie die Mitarbeiter im Rathaus unter 036781-4810 anrufen, wir kümmern uns um Ihre Anliegen.
Das Telefon ist Mo—Do von 7.00—17.00 und freitags von 7.00-12.00 Uhr besetzt.

Ansonsten gelten die bekannten Notrufnummern.
Landratsamt 24 Stunden 03628—738 888
Ärztlicher Bereitschaftsdienst 116 117

Ich möchte Sie dringend darauf hinweisen, dass das Verlassen der Ortschaft Neustadt nicht gestattet ist. Trotz der schwierigen Verhältnisse ist die Versorgung in Neustadt gesichert. So das es keinen Grund gibt, die Ortschaft zu verlassen. Bedenken Sie, dass Sie durch ein solches fahrlässiges Verhalten Ihre Mitmenschen in große Gefahr bringen.

Nur wenn sich alle an die Anweisungen des Landratsamtes des Ilm-Kreises halten, kann schnellstmöglich zu einem normalen Alltag übergegangen werden.

Vermehrt werden jetzt auch die Waldwege kontrolliert. Seien Sie sich sicher, dass Verstöße geahndet und empfindliche Geldstrafen ausgesprochen werden.

Postzustellung

Derzeit werden Post und Pakete von Großbreitenbach an die Übergabestelle nach Neustadt gebracht und dort durch ortsansässige Bürger freiwillig verteilt.
Postsendungen, die gegen Unterschrift zugestellt werden müssen oder Nachnahmen, können während der Quarantäne nicht zugestellt werden.
Post- und Paketversand aus Neustadt ist nicht mehr möglich.

Hundebesitzer

Hundebesitzer, die keine vom Gesundheitsamt festgestellten Kontaktpersonen sind, dürfen Ihre Hunde zum Gassigehen ausführen. Aber bitte nur allein.

Arbeitnehmer

Informieren Sie Ihren Arbeitgeber über die Allgemeinverfügung des Landratsamtes zur erlassenen Quarantäne (weitere Infos siehe Rückseite).

Trotz aller Probleme und Einschränkungen wünsche ich von ganzem Herzen allen Bürgern des Ortsteiles Neustadt und der gesamten Landgemeinde Gesundheit, gute Genesung allen Erkrankten und allen weiteren beste Gesundheit.

Ihr Peter Grimm
Bürgermeister

Veränderte Abfallentsorgung in der Ortschaft Neustadt im Rahmen der Allgemeinverfügung wegen Quarantäne

Aufgrund der Allgemeinverfügung der Landrätin vom 22. März 2020 in Verbindung mit § 25 (4) der Abfallwirtschaftssatzung des Ilm-Kreises vom 15.11.2017 gibt es ab sofort für den Geltungsdauer der Allgemeinverfügung Veränderungen bei der Abfallentsorgung in der Ortschaft Neustadt einschließlich Kahlert der Landgemeinde Stadt Großbreitenbach.

Restabfall

Am 25. März 2020 und 01. April 2020 werden in Neustadt sowie Kahlert die Restabfallbehälter geleert. Die Entsorgung erfolgt wie bisher mittwochs. In den Restabfall müssen auch Bioabfälle und andere Abfälle wie nachfolgend beschrieben eingegeben werden.

Folgende Reglungen sind zu beachten:

- Sämtliche Abfälle, die kontaminiert sein könnten, sollen in stabile, möglichst reißfeste Abfallsäcke gegeben werden. Ein Einwerfen von z.B. losen Taschentüchern in eine Abfalltonne ist zu unterlassen. Die Abfallsäcke sind anschließend durch Verknoten oder Zubinden luftdicht zu verschließen.
- Spitze und scharfe Gegenstände sollen zusätzlich in bruch- und durchstichsicheren Einwegbehältnissen verpackt werden.
- Abfälle mit geringen Mengen Flüssigkeit sollen neben saugfähige Abfälle gelegt werden.
- Es sollen keine Säcke frei zugänglich neben Abfalltonnen oder Container gestellt werden, um Gefahren für Dritte auszuschließen.

Bioabfall

Die getrennte Bioabfallentsorgung wird ausgesetzt. Bitte nutzen Sie die Restabfallgefäße auch für Bioabfall. Bereitgestellte gefüllte Biotonnen werden ausnahmsweise zu den v.g. Terminen mit dem Restabfall entsorgt.

Papiertonnen

Die getrennte Papierentsorgung wird ausgesetzt. Bitte nutzen Sie unbedingt die Restabfallgefäße.

Leichtfraktion: gelbe Tonne und gelbe Säcke:

Die getrennte Sammlung der Leichtfraktion wird ausgesetzt. Bereitgestellte gelbe Säcke oder gelbe Tonnen werden nicht entsorgt. Bitte nutzen Sie unbedingt die Restabfallgefäße.

Sperrmüll

Es erfolgen aktuell keine Abholungen von den Haushaltungen. Bereits zugesagte Termine im Zeitraum der Allgemeinverfügung werden storniert und zu einem späteren Zeitpunkt neu vergeben.

Elektroschrott

Es erfolgen aktuell keine Abholungen von den Haushaltungen. Bereits zugesagte Termine im Zeitraum der Allgemeinverfügung werden storniert und zu einem späteren Zeitpunkt neu vergeben.

Die geplante Elektro-Schrottsammlung am Mittwoch, dem 01. April 2020 findet nicht statt.

Glas und Zentrale Wertstoffsammelplätze

Bei positiver Feststellung einer Infektion sind auch Glasabfälle über die Restmülltonne zu entsorgen. Im Falle der vorsorglichen Quarantäne wird für Glasabfälle und Pfandverpackungen empfohlen, diese nicht über den Hausmüll zu entsorgen, sondern bis zur Aufhebung der Quarantäne im Haushalt aufzubewahren. Eine Reinigung der Oberflächen ist empfehlenswert.

Bitte entsorgen Sie keine Abfälle an den zentralen Standplätzen.

Weitere Informationen erhalten Sie immer aktuell auf der Homepage des AIK.

Wir danken für Ihr Verständnis und stehen bei weiteren Fragen unter Telefon 03628 738-933 und -934 gern zur Verfügung.

Abfallwirtschaftsbetrieb Ilm-Kreis

Merkblatt - Bearbeitung von Entschädigungsanträgen gemäß §§ 56 und 57 Infektionsschutzgesetz (IfSG)

Wer auf Grund des IfSG als Ausscheider, Ansteckungsverdächtiger, Krankheitsverdächtiger oder als sonstiger Träger von Krankheitserregern im Sinne des § 31 Satz 2 IfSG Verboten in der Ausübung seiner bisherigen Erwerbstätigkeit unterliegt oder unterworfen wird und dadurch einen Verdienstausfall erleidet, erhält eine Entschädigung in Geld (§ 56 Absatz 1 IfSG).
Kranke Personen werden vom § 56 IfSG grundsätzlich nicht erfasst, da sie krankheitsbedingt arbeitsunfähig sind. Sie erleiden dadurch keinen Verdienstausfall, da sie entweder Lohnfortzahlung durch den Arbeitsgeber oder Krankengeld durch die Krankenkasse erhalten.
Eltern, deren Kinder wegen eines Besuchsverbots gem. IfSG eine Kindereinrichtung/Schule nicht betreten durften, gehören nicht zu den entschädigungsberechtigten Personenkreisen.
Es wird zusätzlich darauf hingewiesen, dass ein Entschädigungsanspruch nur dann besteht, wenn der Arbeitgeber nicht bereits nach § 616 Abs. 1 BGB zur Entgeltfortzahlung verpflichtet ist. Dies ist grundsätzlich der Fall, es sei denn, dass im geltenden Tarifvertrag oder im individuellen Arbeitsvertrag eine konkrete Regelung getroffen wurde und der Anspruch aus § 616 Abs. 1 BGB ausgeschlossen ist.
Der Antrag ist innerhalb einer **Frist von drei Monaten** nach Einstellung der verbotenen Tätigkeit zu stellen beim:

Thüringer Landesverwaltungsamt
Referat 550 - Gesundheitswesen
Jorge-Semprún-Platz 4
99423 Weimar
Folgendes ist bei Antragstellung zu beachten:

I. Für Arbeitnehmer:
Unterliegen Arbeitnehmer einem Verbot in der Ausübung ihrer bisherigen Erwerbstätigkeit oder werden einem solchem unterworfen und erleiden dadurch einen Verdienstausfall, erhalten sie eine Entschädigung in Geld.
Für die Dauer ihres Arbeitsverhältnisses, längstens für 6 Wochen, hat der Arbeitgeber die Entschädigung an den Arbeitnehmer auszuzahlen. Die ausgezahlten Beträge können dem Arbeitgeber auf Antrag und nach Prüfung der Voraussetzungen erstattet werden.

Folgende Unterlagen sind vom Arbeitgeber einzureichen (§§ 56 u. 57 IfSG):
1. Antragstellung – formlos- mit Angaben der Bankverbindung (IBAN und BIC)
2. Wie lange ist die betroffene Person im Unternehmen bereits beschäftigt?
TLVwA Ref. 550 (Stand: Dez. 2018)
3. Auszug vom Tarifvertrag/Arbeitsvertrag über Regelungen für die Entgeltfortzahlung gem. § 616 Abs. 1 BGB bei Arbeitsausfall, Arbeitsverhinderung, Tätigkeitsverbot und Freistellung von der Arbeit (nicht nur Krankheitsfall) als Kopie beifügen, falls vorhanden.
4. Liegt eine ärztlich attestierte Arbeitsunfähigkeitsbescheinigung wegen Erkrankung für die Zeiten des Tätigkeitsverbotes vor?
Wenn Ja, bitte eine Kopie davon dem Antrag beifügen (Bitte nicht verwechseln mit dem behördlich angeordneten Tätigkeitsverbot des Gesundheitsamtes)
5. Konnte die betreffende Person im Unternehmen umgesetzt werden? Wenn nein, bitte begründen.
6. Angaben über den durchschnittlichen (Jahresdurchschnitt) monatlichen Nettoverdienst sowie der Arbeitgeber- und Arbeitnehmer Anteil zur Rentenversicherung incl. der Vorlage der Lohn- u. Gehaltsabrechnung der letzten 6 Monate.
7. Kopien des behördlich angeordneten Tätigkeitsverbotes des Gesundheitsamtes (Beginn und Ende).

Das komplette Merkblatt können Sie auf der Seite des Gesundheitsministerium unter dem Punkt „Arbeitsrechtliche Informationen" nachlesen.
https://www.tmasgff.de/covid-19
