# Supplementary material for: The role of risk communication in public health interventions. An analysis of risk communication for a community quarantine in Germany to curb the SARS-CoV-2 pandemic
Source: PLoS One. 2021 Aug 13;16(8):e0256113. doi: 10.1371/journal.pone.0256113 (PMC8362954; doi:10.1371/journal.pone.0256113)
Supplement: S3 File — The order contains the announcement of quarantine in Neustadt am Rennsteig. It explains who is affected by quarantine and threatens with consequences in case of breaking the rules. (PDF) [file pone.0256113.s004.pdf]

S3 File. Issued order.

The order contains the announcement of quarantine in Neustadt am Rennsteig. It explains who is affected by quarantine and threatens with consequences in case of breaking the rules.

ILM-KREIS
Die Landrätin
Landratsamt des Ilm-Kreises · Ritterstraße 14 · 99310 Arnstadt

Allgemeinverfügung

Als notwendige Schutzmaßnahmen wird für die gesamte Ortslage Neustadt am Rennsteig der Landgemeinde Stadt Großbreitenbach zum Vollzug des Infektionsschutzgesetzes durch das Landratsamt folgendes angeordnet:

1. Sie haben sich unverzüglich in die häusliche Quarantäne zu begeben.

2. Die gesamte Bevölkerung ist verpflichtet, sich ausschließlich in ihrer Wohnung bzw. auf dem eigenen Wohngrundstücks aufzuhalten.

3. Dies gilt für alle Personen die sich innerhalb der letzten 14 Tage in Neustadt aufgehalten hat.

4. Personen ist der Zutritt bzw. die Zufahrt zur Ortslage nur gestattet, wenn sie dort ihren Haupt- oder Nebenwohnsitz haben.

5. Ausgenommen hiervon sind Rettungsdienste, ärztliche Hausbesuche, medizinische Pflege.

6. Personen, die Erkältungssymptome aufweisen, sind verpflichtet sich unverzüglich telefonisch beim Hausarzt oder dem kassenärztlichen Bereitschaftsdienst unter der Telefonnummer 116 117 zu melden.

7. Alle Personen sind verpflichtet, den direkten Kontakt mit anderen Personen vermeiden.

8. Der Kontakt mit Personen zur Pflege und Versorgung ist auf das absolut notwendige Minimum zu beschränken.

9. Bei medizinischen Behandlungen sind Sie verpflichtet den Rettungsdienst sowie die Arztpraxis oder das Krankenhaus bereits vorab über die Quarantäne und deren Grund zu informieren.

10. Zum Zweck der Selbstversorgung mit lebensnotwendigen Gütern dürfen Personen ohne Erkältungssymptome auf kürzestem Wege die Nahversorgung aufsuchen haben sich im Anschluss unverzüglich wieder in die häusliche Quarantäne zurück zu begeben.

11. Diese Allgemeinverfügung gilt ab sofort bis einschließlich zum 05.04.2020.

12. Zuwiderhandlungen gegen diese Allgemeinverfügung werden strafrechtlich oder als Ordnungswidrigkeit geahndet.

13. Die Allgemeinverfügung kann mit unmittelbarem Zwang durchgesetzt werden.

Petra Enders, Landrätin

Landratsamt des Ilm-Kreises
Ritterstraße 14
99310 Arnstadt
http://www.ilm-kreis.de
Telefon 03628 738-0
Telefax 03628 738-111

Allgemeine Sprechzeiten:
Di. 08:30 - 11:30 Uhr
13:00 - 18:00 Uhr
Do. 08:30 - 11:30 Uhr
13:00 - 14:30 Uhr

Außenstelle Ilmenau
Krankenhausstraße 12a
98693 Ilmenau
Telefon 03677 657-0
Telefax 03677 841075

Allgemeine Sprechzeiten:
Di. 08:30 - 11:30 Uhr
13:00 - 14:30 Uhr
Do. 08:30 - 11:30 Uhr
13:00 - 18:00 Uhr

Bankverbindung:
Sparkasse Arnstadt-Ilmenau
BLZ: 840 510 10
Konto-Nr. 1810000153
BIC: HELADEF1ILK
IBAN:DE79840510101810000153

ILM-KREIS
Die Landrätin
Landratsamt des Ilm-Kreises Ritterstraße 14 99310 Arnstadt

Allgemeinverfügung

Zum Vollzug des Gesetzes zur Verhütung und Bekämpfung von Infektionskrankheiten beim Menschen (Infektionsschutzgesetz - IfSG) ordnet das Landratsamt Ilm-Kreis gemäß § 28 Abs. 1 Satz 1 und 2 IfSG in Verbindung mit § 35 Satz 2 Thüringer Verwaltungsverfahrensgesetz (ThürVwVfG) als notwendige Schutzmaßnahmen an:

Für die gesamte Ortslage der Ortschaft Neustadt am Rennsteig der Landgemeinde Stadt Großbreitenbach werden folgende Maßnahmen angeordnet:

1. Die gesamte Bevölkerung sowohl mit Haupt- als auch Nebenwohnsitz, die sich innerhalb der letzten 14 Tage dort - und sei es auch nur kurzzeitig bzw. vorübergehend - aufgehalten hat ist verpflichtet, sich ausschließlich in ihrer Wohnung bzw. auf ausschließlich selbstgenutzten Bereichen des eigenen Wohngrundstücks aufzuhalten.

2. Personen ist der Zutritt bzw. die Zufahrt zur Ortslage nur gestattet, wenn sie dort ihren Haupt- oder Nebenwohnsitz haben und sich unverzüglich in die häusliche Quarantäne nach Ziffer 1 begeben. Ausgenommen hiervon sind nur Personen die Maßnahmen der medizinisch indizierten Pflege wahrnehmen, Rettungsdienste, ärztliche Hausbesuche und Maß-nahmen vergleichbarer Organisation und Tätigkeiten. Ein Verlassen der Ortslage wird mit Ausnahme des vorstehend besonders genanntem Personenkreises untersagt.

3. Weisen die in Ziffer 1 genannten Personen Erkältungssymptome auf, wie trockener Husten, Fieber, Schnupfen, Abgeschlagenheit, Atemprobleme, sind sie verpflichtet, unverzüglich telefonisch den Hausarzt oder den kassenärztlichen Bereitschaftsdienst unter der Telefonnummer 116 117 zu kontaktieren.

4. Die Personen unter Ziffer 1 sind verpflichtet, den direkten Kontakt mit anderen Personen einzustellen. Der Kontakt mit in häuslicher Gemeinschaft lebenden Personen sowie zur Pflege und Versorgung tätigen Personen ist auf das absolut notwendige Minimum zu reduzieren.

5. Sollte während der angeordneten Quarantänezeit eine medizinische Behandlung erforderlich werden, sind die Personen unter Ziffer 1 und die Personensorgeberechtigten der Personen unter Ziffer 2 verpflichtetet, den Rettungsdienst sowie die sie versorgende medizinische Einrichtung (z.B. Arztpraxis, Krankenhaus) bereits vorab telefonisch über die angeordnete Quarantäne und deren Grund zu informieren.

6. Zum Zweck der Selbstversorgung mit lebensnotwendigen Gütern sind Personen, welche die Erkältungssymptome nach Ziffer 3 nicht aufweisen, berechtigt, auf kürzestem Wege entsprechende Einrichtungen zur Versorgung aufzusuchen. Im Anschluss haben sich diese Personen unverzüglich wieder in die häusliche Quarantäne zurück zu begeben.

7. Diese Allgemeinverfügung gilt ab sofort bis einschließlich zum 05.04.2020. Widerspruch und Anfechtungsklage haben keine aufschiebende Wirkung.

8. Es wird ausdrücklich darauf hingewiesen dass Zuwiderhandlungen gegen diese Allgemeinverfügung strafrechtlich oder als Ordnungswidrigkeit geahndet werden und Maßnahmen dieser Allgemeinverfügung mit unmittelbarem Zwang durchgesetzt werden können.

Rechtsbehelfsbelehrung:
Gegen diese Allgemeinverfügung kann binnen eines Monats nach seiner Bekanntgabe Widerspruch erhoben werden. Der Widerspruch ist entweder schriftlich oder zur Niederschrift beim Ilm-Kreis, Landratsamt, (Gesundheitsamt), Ritterstraße 14, 99310 Arnstadt, einzulegen oder auf elektronischem Wege durch De-Mail in der Sendevariante mit bestätigter sicherer Anmeldung nach dem De-Mail-Gesetz zu erheben. Die De-Mail-Adresse des Ilm-Kreises lautet: poststelle@ilm-kreis.de-mail.de.
Die Frist ist auch gewahrt, wenn der Widerspruch beim Thüringer Landesverwaltungsamt, Jorge-Semprún-Platz 4, 99423 Weimar, eingelegt wird.

Hinweise:
Die Anordnungen sind auch dann zu befolgen, wenn hiergegen ein Rechtsbehelf (Widerspruch, Anfechtungsklage) eingelegt wird. Widerspruch und Anfechtungsklage haben gemäß § 28 Abs. 3 IfSG i.V.m. § 16 Abs. 8 IfSG i.V.m. § 80 Abs. 2 Satz 1 Nr. 3 Verwaltungsgerichtsordnung (VwGO) keine aufschiebende Wirkung.
Die Anordnung der aufschiebenden Wirkung eines Widerspruchs kann beim Verwaltungsgericht Weimar, Jenaer Straße 2, 99425 Weimar, beantragt werden.

Auf die Bußgeldvorschriften des § 73 Abs. 1a Nr. 6 IfSG wird hingewiesen.
Durch die Verordnung über die Ausdehnung der Meldepflicht nach § 6 Absatz 1 Satz 1 Nr. 1 und § 7 Absatz 1 Satz 1 des Infektionsschutzgesetzes auf Infektionen mit dem erstmals im Dezember 2019 in Wuhan/Volksrepublik China aufgetretenen neuartigen Coronavirus ("2019-nCoV") (CoronaMeldeV), die am 01.02.2020 in Kraft getreten ist, wurde die Meldepflicht nach § 6 Abs. 1 Satz 1 Nr. 1 IfSG auf den Verdacht einer Erkrankung, die Erkrankung sowie den Tod in Bezug auf eine Infektion, die durch 2019-nCoV (SARS-CoV-2) hervorgerufen wird, sowie nach § 7 Absatz 1 Satz 1 IfSG auf den direkten oder indirekten Nachweis von 2019-nCoV (SARS-CoV-2), soweit der Nachweis auf eine akute Infektion hinweist, ausgedehnt.
Damit sind die Strafvorschriften des § 74 IfSG anwendbar.

Aufgrund der Gefahr im Verzug wurde der Verwaltungsakt heute am 22.03.2020 mündlich durch Lautsprecherdurchsagen bekanntgegeben. Der schriftliche Teil wird durch Aushang im Landratsamt Ilm-Kreis bekanntgegeben. Gemäß § 41 Abs. 4 Satz 1 ThürVwVfG ist nur der verfügende Teil der Allgemeinverfügung öffentlich bekannt zu machen. Die Begründung wird durch Aushang im Landratsamt Ilm-Kreis, Ritterstraße 14, 99310 Arnstadt, veröffentlicht.

Arnstadt, 22.03.2020

THÜRINGEN ILM-KREIS 2

Petra Enders
Landrätin

(Siegel)
