# Supplementary material for: The role of risk communication in public health interventions. An analysis of risk communication for a community quarantine in Germany to curb the SARS-CoV-2 pandemic
Source: PLoS One. 2021 Aug 13;16(8):e0256113. doi: 10.1371/journal.pone.0256113 (PMC8362954; doi:10.1371/journal.pone.0256113)
Supplement: Attachment — Submitted filename: summary open-ended questions.docx [file pone.0256113.s005.docx]

**Community quarantine - understanding communities and public health risk communication in the management of the Sars-CoV-2 pandemic**

**Summary**

Non-pharmaceutical public health interventions require the cooperation and compliance of the affected communities. Quarantine orders pose a particularly difficult communication challenge because the persons affected, which are healthy individuals that are suspected to become contagious, have to remain physically separated from their friends and family and are confined to their own space. While there is a growing body of the psychological impacts of quarantine on individuals and communication advice to public health officials, there is little evidence on how to implement and communicate quarantine orders that affect entire communities or villages.

During the first wave of the Sars-CoV-2 pandemic, we investigated a community quarantine that was imposed on a small village (less than 900 residents) in a rural area in Germany (Thuringia) with the aim to better understand the information and communication requirements of the community and to gain insights on how public health officials can improve their communication.

This study was comprised of a questionnaire that included quantitative and qualitative methodologies for analysis. The summary of the qualitative analysis is focused around the three pillars of risk communication and stresses:

*Information*

1) The importance of a transparent flow of information;
2) The physical presence or approachability of a decision maker (tangible leadership);
3) The provision of an overview or process information about the intervention

*Communication*

1) Communication between decision-makers and residents were rare, but constantly requested by residents.
2) Communication among peers, partners and family (horizontal communication) played an important role.
3) The use of social media such as WhatsApp was high, but hardly used for official communication.

*Coordination*

1) The community condemned deviant behaviour, such as violating quarantine orders.
2) Places or people with a high contamination risk were avoided by residents (such as the local supermarket).

[author’s note: The qualitative analysis concerns the open-ended questions 6, 8, 10, 14, 15, 16 of the questionnaire which can be found in the Supplementary Material (in German).]
